# Combined Effects of Genetic Variants of the *PTEN*, *AKT1*, *MDM*2 and *p53* Genes on the Risk of Nasopharyngeal Carcinoma

**DOI:** 10.1371/journal.pone.0092135

**Published:** 2014-03-14

**Authors:** Xiaoai Zhang, Xi Chen, Yun Zhai, Ying Cui, Pengbo Cao, Hongxing Zhang, Zhihao Wu, Peiyao Li, Lixa Yu, Xia Xia, Fuchu He, Gangqiao Zhou

**Affiliations:** 1 State Key Laboratory of Proteomics, Beijing Proteome Research Center, Beijing Institute of Radiation Medicine, Beijing, P. R. China; 2 Affiliated Cancer Hospital of Guangxi Medical University, Nanning, P. R. China; 3 State Key Laboratory of Pathogen and Biosecurity, Beijing Institute of Microbiology and Epidemiology, Beijing, P. R. China; MOE Key Laboratory of Environment and Health, School of Public Health, Tongji Medical College, Huazhong University of Science and Technology, China

## Abstract

Phosphatase and tensin homolog (PTEN), v-akt murine thymoma viral oncogene homolog 1 (AKT1), mouse double minute 2 (MDM2) and p53 play important roles in the development of cancer. We examined whether the single nucleotide polymorphisms (SNPs) in the *PTEN*, *AKT1*, *MDM2* and *p53* genes were related to the risk and severity of nasopharyngeal carcinoma (NPC) in the Chinese population. Seven SNPs [*p53* rs1042522, *PTEN* rs11202592, *AKT1* SNP1-5 (rs3803300, rs1130214, rs3730358, rs1130233 and rs2494732)] were genotyped in 593 NPC cases and 480 controls by PCR direct sequencing or PCR-RFLP analysis. Multivariate logistic regression analysis was used to calculate adjusted odds ratios (ORs) and 95% confidence intervals (CIs). None of the polymorphisms alone was associated with the risk or severity of NPC. However, haplotype analyses indicated that a two-SNP core haplotype (SNP4-5, AA) in *AKT1* was associated with a significantly increased susceptibility to NPC risk (adjusted OR  =  3.87, 95% CI  =  1.96–7.65; *P*<0.001). Furthermore, there was a significantly increased risk of NPC associated with the combined risk genotypes (i.e., *p53* rs1042522 Arg/Pro + Pro/Pro, *MDM2* rs2279244 G/T + G/G, *PTEN* rs11202592 C/C, *AKT1* rs1130233 A/A). Compared with the low-risk group (0–2 combined risk genotypes), the high-risk group (3-4 combined risk genotypes) was associated with a significantly increased susceptibility to NPC risk (adjusted OR  =  1.67, 95% CI  =  1.12–2.50; *P* = 0.012). Our results suggest that genetic variants in the PTEN, AKT1, MDM2 and p53 tumor suppressor-oncoprotein network may play roles in mediating the susceptibility to NPC in Chinese populations.

## Introduction

Nasopharyngeal carcinoma (NPC) is a rare malignancy in most parts of the world, but it occurs at relatively high rates in some geographic regions and among certain ethnic groups. According to global cancer statistics from the International Agency for Research on Cancer, there were over 84,000 incident cases of NPC and 51,600 deaths in 2008, with 80% of the cases located in Asia [1], [2]. The disease is a major public health challenge in southeast China, where it accounts for 20% of all cancers [3], [4]. Over the years, numerous studies have revealed that NPC is a complex disease caused by the interaction of Epstein-Barr virus (EBV) infection, environmental and host genetics factors in a multi-step process of carcinogenesis [5]. Currently available data on the origin of NPC suggests that the genetic alterations of tumor suppressor genes and oncogenes may be important in NPC carcinogenesis.

The phosphatase and tensin homolog (PTEN), v-akt murine thymoma viral oncogene homolog 1 (AKT1), mouse double minute 2 (MDM2) and p53 tumor suppressor-oncoprotein network plays a crucial role in regulating a number of cellular processes such as cell growth, apoptosis, survival and cell cycle, which ultimately contributes to cancer development and progression [6]–[8]. The p53 tumor suppressor protein plays a central role in the prevention of tumor development, and tumorigenesis is accelerated when p53 activity is inhibited [9], [10]. Recent observations demonstrated that AKT1 phosphorylates and stabilizes MDM2, the principal negative regulator of p53, resulting in the downregulation of p53 activity [11]. On the other hand, AKT1 is negatively regulated by PTEN, a p53 response gene that is inactivated in a variety of cancers [12]. Thus, two known tumor suppressor proteins (p53 and PTEN) and two oncoproteins (MDM2 and AKT1) are networked to balance cell survival and apoptosis [7]. The gain and loss of function of the oncogenic and tumor suppressor components of this network have been extensively described in a variety of cancers [7], [13] including NPC [14]–[17]. Because of the importance of this tumor suppressor-oncoprotein network in cancer development and progression, we hypothesized that genetic variations within this network may lead to the deregulation of proliferation or cell death and subsequently affect cancer risk.

Several single nucleotide polymorphisms (SNPs) in the *PTEN*, *AKT1*, *MDM2* and *p53* genes have been well characterized. The *p53* gene has a single base change of G to C at codon 72 in exon 4, known as the *p53* Arg72Pro polymorphism (rs1042522), which causes alteration of amino acid residue from arginine to proline. The p53 Pro72 allele is weaker than the Arg72 allele in inducing apoptosis and suppressing cellular transformation, but it appears to be better at initiating senescence and cell cycle arrest [18]–[20]. Of the identified *MDM2* variants, the SNP309 polymorphism (rs2279244) is a T to G change at nucleotide 309 in the first intron. Compared with the T allele, the G allele has been shown to result in the increased expression of *MDM2* RNA and protein and the subsequent down regulation of the p53 pathway [21], [22]. Recent studies indicate that several specific combinations of SNPs in the *AKT1* gene have been associated with variable AKT1 expression and p53-dependent apoptosis [23]. In addition, a polymorphism located in the 5′ untranslated region of the *PTEN* gene, C-9G (rs11202592), was shown to result in enhanced PTEN expression, which subsequently led to a reduced insulin-induced phosphorylation of AKT [24]. Taken together, these data indicated that the PTEN, AKT1, MDM2 and p53 tumor suppressor-oncoprotein network is genetically heterogeneous, a feature that can lead to a wide variation in the p53 response and may ultimately influence cancer risk [25].

We have previously reported that the *MDM2* SNP309 polymorphism is associated with increased susceptibility and advanced lymph node metastasis to NPC [26]. Given the role of the PTEN, AKT1, MDM2 and p53 tumor suppressor-oncoprotein network in the regulation of cell survival and apoptosis, we hypothesize that the combined genetic variants in this tumor suppressor-oncoprotein network could collectively modify the risk of NPC; and these combined risk genotypes could serve as susceptibility markers for identifying high-risk subgroups of patients who might benefit from personalized prevention and treatment. Therefore, in this study, we investigated the combined effects of genetic variants in the *PTEN, AKT1*, *MDM2* and *p53* genes on the risk and disease severity of NPC in the Chinese population.

## Materials and Methods

### Ethics statement

The study was performed with the approval of the Ethical Committee of Beijing Institute of Radiation Medicine and conducted according to the principles expressed in the Declaration of Helsinki. Written informed consent was obtained from all the participants before inclusion in the study.

### Study subjects

This case-control study consisted of 593 patients with NPC and 480 controls that have been described previously [26]. All subjects were unrelated ethnic Chinese and were enrolled from Nanning city and its surrounding regions between September 2003 and July 2005. The diagnosis of cases, the inclusion and exclusion criteria for cases and controls, the definition of smokers and drinkers, and the tumor staging were described previously [26]. At the time of recruitment, personal information including demographic factors, medical history, tobacco and alcohol use, and family history of NPC were collected via a structured questionnaire.

### Genotype analysis

Genomic DNA from peripheral blood was isolated by using standard phenol/chloroform protocols. The *p53* Arg72Pro polymorphism (rs1042522) was genotyped using polymerase chain reaction (PCR) direct sequencing. Polymorphisms in the *PTEN* (C-9G, rs11202592) and *AKT1* (SNP1, rs3803300; SNP2, rs1130214; SNP3, rs3730358; SNP4, rs1130233; and SNP5, rs2494732) genes were genotyped using PCR-based restriction fragment length polymorphism (RFLP) analysis. The primers and the restriction enzymes used in the study are listed in Table 1. PCR conditions were identical to those used for the SNP discovery described previously [27]. Genotyping was performed by staff blinded to the subjects’ case/control statuses. The accuracy of the genotyping data for each polymorphism obtained from PCR-RFLP analyses was validated by direct DNA sequencing of a 15% masked, random sample of cases and controls.

**Table 1:** Primers and restriction enzymes used to investigate polymorphisms in the *PTEN*, *AKT1* and *p53* genes.

Genes	Polymorphisms	Primer sequences^a^	Methods	Restriction enzymes	
*p53*	Arg72Pro (rs1042522)	Forward: 5′-CCGGAACTGCTTCCTGTCTT-3′	PCR-sequencing		
		Reverse: 5′-CTCCATCTGCCTCCAGAACC-3′			
*PTEN*	C-9G (rs11202592)	Forward: 5′-CAAGTCCAGAGCCATTTCCATT-3′	PCR-RFLP	*Ava*II	
		Reverse: 5′-GGACATTTTCGCATCCGTCTA-3′			
*AKT1*	SNP1 (rs3803300)	Forward: 5′-CTCCATCTGCCTCCAGAACC-3′	PCR-RFLP	*Alw26*I	
		Reverse: 5′-GCTGTGGCATCATTGTCACTC-3′			
	SNP2 (rs1130214)	Forward: 5′-GTGCTCCTCACTGACGGACTT-3′	PCR-RFLP	*Xcm*I	
		Reverse: 5′-AGCCTCCCTAACCTGATGCAC-3′			
	SNP3 (rs3730358)	Forward: 5′-AACAACTTCTCTGTGGCGCGTAAGTATCCCCT*a*GGC-3′	PCR-RFLP	*Stu*I	
		Reverse: 5′-GCCTTAGGACTCAGCCTGGA-3′			
	SNP4 (rs1130233^b^)	Forward: 5′-CTGCTGTGGGGTGACTTGTTC-3′	PCR-RFLP	*HpyCH4*IV	
		Reverse: 5′-AGGTAGTCCAGGGCTGACACA-3′			
	SNP5 (rs2494732)	Forward: 5′-CCCAAGCACGTCACACCTC-3′	PCR-RFLP	*Pst*I	
		Reverse: 5′-GGGACAGAGGCCCAACTGAC-3′			

a Italicized lowercase letters indicate base mismatches.

b Designated rs2498799 in previous literature.

### Statistical analysis

The genotype and allele frequencies for the polymorphisms were determined by gene counting. The fitness to Hardy-Weinberg equilibrium was tested using the random-permutation procedure implemented in the Arlequin package (available at http://lgb.unige.ch/arlequin/). Associations between polymorphisms and the risk of NPC were estimated by logistic regression analyses and adjusted for confounding factors (including age, sex, smoking and drinking status, smoking level, and nationality). Odds ratios (ORs) and 95% confidence intervals (CIs) were used to measure the strength of the association. The potential modification effect of the polymorphism on NPC risk was assessed for the above confounding factors by the addition of interaction terms in the logistic model and by separate analyses of subgroups of subjects stratified by these factors. A homogeneity test was used to compare the difference of ORs within each stratum. In view of the multiple comparisons, the correction factor n (m–1) (n loci with m alleles each) was applied to correct the significance level. These analyses were performed using SPSS software (version 11.5; SPSS Inc.). The pairwise linkage disequilibrium (LD) calculation (Lewontin’s *D* ´ and *r* ^2^) and haplotype blocks construction were performed using the program HaploView 4.2 [28]. Haplotypes based on the polymorphisms in the *AKT1* gene were inferred using PHASE 2.1 software (available at http://www.stat.washington.edu/stephens/). Haplotype frequencies of the cases and controls were compared using *χ* ^2^ tests. The haplo.glm program (available at http://rss.acs.unt.edu/Rdoc/library/haplo. stats/html/haplo.glm.html) was then used to calculate adjusted ORs for each haplotype, and the number of simulations for empirical *P* values was set at 1000.

## Results

### Individual polymorphisms and the risk of NPC

The genotyping results of the seven polymorphisms are presented in Table 2. The observed genotype frequencies for seven polymorphisms were in Hardy-Weinberg equilibrium (all *P*>0.05, data not shown). The genotype frequencies of all seven SNPs among patients were not significantly different from those among the controls. Further, on the basis of logistic regression analysis with adjustment for age, sex, smoking and drinking status, smoking level, and nationality, we found no association with the risk of NPC for these polymorphisms. The associations between the seven polymorphisms and the risk of NPC were further examined with stratification by age, sex, smoking and drinking status, smoking level and nationality. Again, no significant association was found (data not shown). The effect of the seven polymorphisms on the severity of NPC, as measured by the tumor-node-metastasis (TNM) staging system, was also assessed. However, the distributions of genotypes of these polymorphisms were not significantly different among the subgroups with different clinical stage, or different T, N and M classification of the cancer (data not shown).

**Table 2:** The genotype frequencies of polymorphisms in the *PTEN*, *AKT1* and *p53* genes in patients with nasopharyngeal carcinoma and controls.

Genes	SNPs and genotypes	Cases, n (%)	Controls, n (%)	OR (95% CI)^a^	*P* a
*p53*	Arg72Pro				
	Arg/Arg	133 (23.5)	130 (27.2)	Reference	
	Arg/Pro	292 (51.6)	229 (48)	1.25 (0.92–1.70)	0.15
	Pro/Pro	141 (24.9)	118 (24.7)	1.24 (0.87–1.76)	0.23
	Arg/Pro + Pro/Pro	433 (76.5)	347 (72.8)	1.25 (0.94–1.66)	0.13
*PTEN*	C-9G				
	C/C	523 (92.4)	420 (88.2)	Reference	
	C/G	43 (7.6)	56 (11.8)	0.74 (0.48–1.14)	0.17
	G/G	0 (0.0)	0 (0.0)	NA	NA
	C/G + G/G	43 (7.6)	56 (11.8)	0.74 (0.48–1.14)	0.17
*AKT1*	SNP1				
	A/A	329 (60.9)	310 (64.7)	Reference	
	A/G	188 (34.8)	148 (30.9)	1.20 (0.91–1.57)	0.20
	G/G	23 (4.3)	21 (4.4)	1.03 (0.55–1.92)	0.94
	A/G + G/G	211 (39.1)	169 (35.3)	1.17 (0.90–1.52)	0.23
	SNP2				
	G/G	464 (86.7)	425 (88.7)	Reference	
	G/T	68 (12.7)	52 (10.9)	1.15 (0.78–1.71)	0.48
	T/T	3 (0.6)	2 (0.4)	1.01 (0.16–6.43)	0.99
	G/T + T/T	71 (13.3)	54 (11.3)	1.16 (0.79–1.71)	0.45
	SNP3				
	C/C	491 (85.7)	394 (82.1)	Reference	
	C/T	77 (13.4)	80 (16.7)	0.69 (0.20–2.28)	0.52
	T/T	5 (0.9)	6 (1.2)	0.86 (0.24–3.01)	0.81
	C/T + T/T	82 (14.3)	86 (17.9)	0.77 (0.55–1.08)	0.14
	SNP4				
	A/A	281 (50.6)	230 (49.1)	Reference	
	A/G	224 (40.4)	200 (42.7)	0.91 (0.70–1.19)	0.50
	G/G	50 (9)	38 (8.2)	1.06 (0.66–1.69)	0.81
	A/G + G/G	274 (49.4)	238 (50.9)	0.94 (0.73–1.20)	0.61
	SNP5				
	G/G	322 (60.8)	291 (61.1)	Reference	
	G/A	172(32.1)	168 (35.2)	0.93 (0.69–1.22)	0.56
	A/A	36 (6.8)	18 (3.8)	1.79 (0.96–3.39)	0.05
	G/A + A/A	208 (39.2)	186 (39.0)	1.01 (0.77–1.30)	0.91

NOTE: The number of samples that were genotyped varies due to genotyping failure for some individuals.

Abbreviations: OR, odds ratio; CI, confidence interval; NA, not applicable.

a ORs and *P* values were adjusted for age, sex, smoking and drinking status, smoking level and nationality.

### Haplotypes and risk of NPC

**Figure 1:**
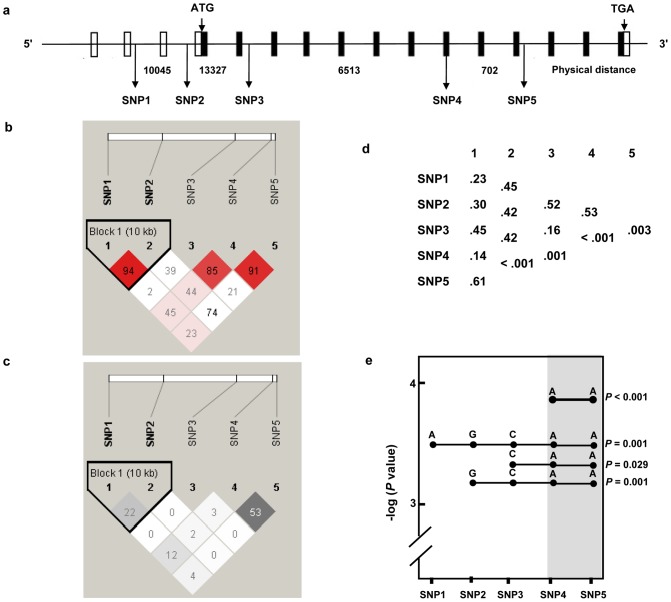
Haplotypes of *AKT1* polymorphisms and the risk of nasopharyngeal carcinoma. (*a*) Genomic structure of the *AKT1* locus and the polymorphic sites used. Exons (boxes) and introns are not drawn to scale; open boxes represent noncoding sequences, and filled boxes represent coding sequences. The physical distance between SNPs is shown in nucleotides. (*b*) Linkage disequilibrium (LD) map of SNPs based on *D* ´. (*c*) LD map of SNPs based on *r* ^2^. (*d*) Global *P* values from single-locus and multi-locus (two to five) based association analysis. (*e*) Haplotypes showing significant genetic associations with the risk of nasopharyngeal carcinoma. The two-SNP core haplotype is highlighted in gray.

The pairwise disequilibria measures (*D* and *r* ^2^) of the five *AKT1* polymorphisms were calculated. Figure 1b and 1c showed that two polymorphisms, SNP4 and SNP5, were in strong LD. We next performed haplotype analysis to derive haplotypes specifically correlated with NPC. We identified several multi-SNP haplotype systems (Figure 1d). Further multilocus analysis identified a two-SNP haplotype (SNP4-5, AA) that was associated with significant increased susceptibility to NPC risk (adjusted OR  = 3.87, 95% CI  = 1.96–7.65; *P*<0.001) (Figure 1e). Three other multi-SNP haplotypes (SNP1-5, AGCAA; SNP3-5, CAA; SNP2-5, GCAA) also showed a significant association with NPC risk; they all share a common core that extends from SNP4 to SNP5 (Figure 1e).

The effect of the haplotypes on severity of NPC was also assessed. However, the distributions of haplotype frequencies were not significantly different among the subgroups with different clinical stages, or different T, N and M classification of the cancer (data not shown).

### Combined effects of the genetic variants on risk of NPC

Considering that each of these polymorphisms appeared to have a weak effect on NPC risk, we next investigated the combined effects of three functional polymorphisms (*MDM2* SNP309, *p53* Arg72Pro and *PTEN* C-9G) and two associated polymorphisms (*AKT1* SNP4 and SNP5) on NPC risk. Because the *AKT1* SNP4 and SNP5 were in strong LD, we only included *AKT1* SNP4 in the analysis. Specifically, in the study subjects who had data available for all four polymorphisms, we categorized all risk genotypes of each polymorphism (i.e., *p53* rs1042522 Arg/Pro + Pro/Pro, *MDM2* rs2279244 G/T + G/G, *PTEN* rs11202592 C/C, *AKT1* rs1130233 A/A) into a new variable according to the number of risk genotypes carried by an individual. When we combined the risk genotypes of the four polymorphisms together, we found that the risk for NPC increased significantly as the number of the combined risk genotypes increased (*P*_trend_  =  0.019). We then categorized the patients into two groups: (i) the low-risk group (0-2 combined risk genotypes) and (ii) the high-risk group (3–4 combined risk genotypes). The frequencies of the combined risk genotypes among the cases were significantly different from those among the controls (*P* = 0.005). Furthermore, using the low-risk group as the reference group, the high-risk group was significantly associated with an increased susceptibility to NPC risk (adjusted OR  =  1.67, 95% CI  =  1.12–2.50; *P* = 0.012; Table 3). The association remained significant even after correction for multiple comparisons (*P* = 0.048).

**Table 3:** Combined effects of the genetic variants in the *PTEN*, *AKT1*, *MDM2* and *p53* genes on the risk of nasopharyngeal carcinoma.

	Cases, n (%)	Controls, n (%)	*P*	OR (95% CI)	*P* b
No. of risk genotypes			0.017^a^		
0–2 risk genotypes	48 (9.2)	68 (15.0)		Reference	
3 risk genotypes	210 (40.2)	175 (38.7)		1.62 (1.06–2.49)	0.026
4 risk genotypes	265 (50.7)	209 (46.2)		1.71 (1.11–2.61)	0.013
*P*_trend_			0.019^c^		
Combined risk genotypes^d^			0.005^a^		
Low-risk group	48 (9.2)	68 (15.0)		Reference	
High-risk group	475 (90.8)	384 (85.0)		1.67 (1.12–2.50)	0.012*

Abbreviations: OR, odds ratio; CI, confidence interval.

a *χ^2^* test for the distribution of genotypes between patients and control subjects.

b *P* values were calculated by multivariate logistic regression, adjusted for age, sex, smoking and drinking status, smoking level, and nationality.

c *χ* ^2^ test for the *P*_trend_ value of genotypes between patients and control subjects.

d Low-risk group, individuals carrying 0–2 risk genotypes; high-risk group, individuals carrying 3-4 risk genotypes.

*P value remained significant after c°rrection for multiple comparisons (*P* = 0.048).

We also evaluated the association between the combined risk genotypes and risk of NPC stratified by age, sex, smoking and drinking status, smoking level, and nationality (Table 4). Although the susceptibility to NPC seemed to be more pronounced in subjects who were male, older (> 47 years), nonsmokers and those of Han nationality, these differences could be attributed to chance (all *P*>0.05, test for homogeneity), indicating that these potential confounding factors had no modification effect on the risk of NPC.

**Table 4:** Stratification analysis of the combined genotypes of the *PTEN*, *AKT1*, *MDM2* and *p53* polymorphisms and risk of nasopharyngeal carcinoma.

Variables	Cases, n (%)		Controls, n (%)		*P* a	OR (95% CI)^a^	*P*_Homogeneity_ b
	Low-risk group^c^	High-risk group^c^	Low-risk group^c^	High-risk group^c^			
Sex							
Male	38 (10.1)	337 (89.9)	57 (15.7)	307 (84.3)	0.026	1.65 (1.06–2.58)	0.85
Female	10 (6.8)	138 (93.2)	11 (12.5)	77 (87.5)	0.31	1.64 (0.62–4.32)	
Age (year)							0.62
≤ 47	22 (8.1)	250 (91.9)	23 (10.8)	191 (89.2)	0.46	1.28 (0.66–2.47)	
> 47	26 (10.4)	225 (89.6)	45 (18.9)	193 (81.1)	0.01	1.97 (1.16–3.33)	
Smoking status							0.97
Nonsmoker	32 (8.9)	328 (91.1)	46 (15.1)	259 (84.9)	0.036	1.68 (1.03–2.73)	
Smoker	16 (9.8)	147 (90.2)	22 (15)	125 (85)	0.12	1.81 (0.86–3.81)	
Drinking status							0.57
Nondrinker	28 (8.8)	290 (91.2)	44 (13.9)	273 (86.1)	0.072	1.60 (0.96–2.69)	
Drinker	20 (9.8)	185 (90.2)	24 (17.8)	111 (82.2)	0.06	1.90 (0.97–3.71)	
Smoking level (pack-years)							0.36
≤ 19	5 (9.1)	50 (90.9)	11 (12.9)	74 (87.1)	0.34	1.83 (0.53–6.36)	
> 19	11 (10.2)	97 (89.8)	11 (17.7)	51 (82.3)	0.15	2.03 (0.78–5.31)	
Nationality							0.71
Han	33 (8.9)	338 (91.1)	51 (15.3)	283 (84.7)	0.008	1.90 (1.17–3.07)	
Non-Han	15 (9.9)	137 (90.1)	17 (14.4)	101 (85.6)	0.61	1.25 (0.53–2.90)	

Abbreviations: OR, odds ratio; CI, confidence interval.

a ORs and *P* values were calculated by multivariate logistic regression, adjusted for age, sex, smoking and drinking status, smoking level and nationality when appropriate within the strata.

b For differences in ORs within each stratum.

c Low-risk group, individuals carrying 0–2 risk genotypes; high-risk group, individuals carrying 3–4 risk genotypes.

Furthermore, we evaluated the effect of combined risk genotypes on the severity of NPC. However, the distributions of frequencies of combined risk genotypes were not significantly different among the subgroups with different clinical stage or different T, N and M classification of the cancer (data not shown).

## Discussion

The PTEN, AKT1, MDM2 and p53 tumor suppressor-oncoprotein network plays an important role in the development of cancers. Polymorphisms within this network may affect their corresponding protein expression or function and, thus, potentially affect the risk to developing various cancers. However, the role of genetic variations of this tumor suppressor-oncoprotein network in NPC is not yet fully understood. In this study, we found that genetic variants of the *PTEN*, *AKT*1, *MDM2* and *p53* genes jointly influence the susceptibility to NPC risk. These results suggest that genetic variation within the PTEN, AKT1, MDM2 and p53 network can be used as biomarkers to identify high-risk subgroups of patients who might benefit from personalized prevention and treatment.

The genetic associations observed in this study are biologically plausible. P53, MDM2, PTEN and AKT1 each have a role in carcinogenesis and tumor progression. The deregulation of these four genes has been detected in a broad range of human malignancies including NPC [13]–[17]. Furthermore, p53, MDM2, PTEN and AKT1 can interact with each other to balance cell survival and apoptosis. The major role of MDM2 is to interact directly with p53 to block p53-mediated transactivation and apoptosis. AKT1 is an antiapoptotic protein kinase, and one of its substrates is MDM2 protein. The phosphorylation of MDM2 by AKT1 leads to the stabilization of MDM2 and also promotes the movement of MDM2 into the nucleus where it can act to downregulate p53 activity [11]. On the other hand, the major function of PTEN relies on its AKT1 inhibitory activity, and the loss of PTEN function results in increased AKT1 activation [12]. In addition, PTEN can directly inhibit the movement of MDM2 into the nucleus, thereby protecting p53 from survival signals emanating from growth factor receptors [29]. Therefore, these numerous interactions may support the biological plausibility that the combination of variants of the PTEN, AKT1, MDM2 and p53 network could result in more comprehensive and accurate estimates of risk for NPC than can be obtained from a single variant.

Another finding in the present study was that a two-SNP core haplotype in the *AKT1* gene, SNP4-5 AA, was significantly associated with increased NPC risk. AKT1 is a central node in cell signaling that plays an important role in tumorigenesis. AKT1 has been reported to be constitutively activated in NPC, enhancing cell survival by blocking the induction of apoptosis [15]. Haplotypes in the *AKT1* gene were recently reported to be associated with higher levels of AKT1 protein expression and are resistant to p53-dependent apoptosis [23], [30]. One study also reported an association between the *AKT1* polymorphism and cancer metastasis [31]. Additionally, *AKT1* polymorphisms were found to predict treatment response and clinical outcome in patients with esophageal and non-small cell lung cancer [32], [33]. Collectively, these observations indicate that our finding of an association between the *AKT1* haplotype and the risk of NPC may be biologically plausible.

The molecular mechanism by which the *AKT1* SNP4-5 AA haplotype confers a risk of developing NPC is unknown. It has not been shown that either SNP4 (a silent change at amino acid 242 in exon 11) or SNP5 (located in intron 13) represent a functional SNP with an ability to change either the expression or activity of AKT1. Rather, these two SNPs may only be markers for this region, and a unique variant capturing the effect of both SNP4 and SNP5 remains to be discovered. In addition, Emamian et al. reported that a core risk haplotype TC, extending from *AKT1* SNP2 to SNP3, was associated with lower AKT1 protein levels in EBV-transformed lymphocytes in Americans of Northern European descent [30]. However, Harris et al. demonstrated that B cells harboring the major SNP3-4 haplotype at *AKT1* expressied higher levels of AKT1and are relatively resistant to p53-dependent apoptosis compared to cells with the minor haplotype in Caucasians [23]. The inconsistency between these findings may be due to the difference of LD between a functional SNP and a marker SNP in different populations. Indeed, the LD patterns and allele frequencies in this region vary in different racial populations. Thus, there may be “race-specific” differences in the contribution of polymorphisms to AKT1 expression and, consequently, to cancer risk. However, additional studies are needed to clarify this possibility.

Polymorphisms in the PTEN, AKT1, MDM2 and p53 network have been individually used to search for susceptibility alleles of different cancers, but the results are inconsistent. The inconsistent results of these studies may be attributed to different molecular mechanisms of carcinogenesis among cancers, small sample size, marginal statistical significance and different ethnicities of the study populations. Additionally, a minor effect of a single variant on cancer risk could also cause the inconsistent results. Several studies have reported a potential interaction between the *MDM2* SNP309 and the *p53* Arg72Pro polymorphisms for breast and endometrial cancer, gastric cardia adenocarcinoma, and hepatitis B virus-related hepatocellular carcinoma [34]–[37]. Interactions were observed between the *p53* Arg72Pro and *PTEN* polymorphisms with regard to the risk of esophageal squamous cell carcinoma [38]. This is particularly true in the present study, in which the haplotype and combined analyses confirmed the effects of multi-SNPs on NPC risk. Our results, together with those of the earlier studies, highlight the need for the combined analysis of genetic variants on cancer risk.

In reviewing the results of this study, one must also keep several potential limitations in mind. First, as a hospital-based study, our cases were enrolled from the hospitals and the controls were selected from the community population. Consequently, inherent selection bias might have occurred. To overcome this limitation, we matched cases and controls for their age and residential area. Moreover, any inadequacy in matching was controlled in the data analyses with further adjustment and stratification. Second, considering our study population included a small number of patients with the low-risk genotype group, our initial findings should be investigated in additional studies with larger sample sizes. Third, in this study, we selected variants from four genes that encode the core functional components of this tumor suppressor-oncoprotein network. However, this network is complex, and further studies that investigate other genes in this network are warranted to fully clarify the role of this important tumor suppressor-oncoprotein network in the genetic etiology of cancers.

In summary, to our knowledge, this report is the first to describe the association between the combined effects of genetic variants of the PTEN, AKT1, MDM2 and p53 tumor suppressor-oncoprotein network and the risk of NPC. If confirmed by other studies, the contribution of genetic factors to the pathogenesis of the NPC presented here may have implications for the screening and treatment of this disorder.
